# Design of a Current-Mode OTA-Based Memristor Emulator for Neuromorphic Medical Application

**DOI:** 10.3390/mi16080848

**Published:** 2025-07-24

**Authors:** Amel Neifar, Imen Barraj, Hassen Mestiri, Mohamed Masmoudi

**Affiliations:** 1 Systems Integration & Emerging Energies (SI2E), Electrical Engineering Department, National Engineering School of Sfax, University of Sfax, Sfax 3038, Tunisia; 2 Department of Computer Engineering, College of Computer Engineering and Sciences, Prince Sattam Bin Abdulaziz University, Al-Kharj 11942, Saudi Arabia

**Keywords:** memristor emulator, operational transconductance amplifier, CMOS, Memristive Integrate-and-Fire, circuit neuron, retinal protheses

## Abstract

This study presents transistor-level simulation results for a novel memristor emulator circuit. The design incorporates an inverter and a current-mode-controlled operational transconductance amplifier to stabilize the output voltage. Transient performance is evaluated across a 20 MHz to 100 MHz frequency range. Simulations using 0.18 μm TSMC technology confirm the circuit’s functionality, demonstrating a power consumption of 0.1 mW at a 1.2 V supply. The memristor model’s reliability is verified through corner simulations, along with Monte Carlo and temperature variation tests. Furthermore, the emulator is applied in a Memristive Integrate-and-Fire neuron circuit, a CMOS-based system that replicates biological neuron behavior for spike generation, enabling ultra-low-power computing and advanced processing in retinal prosthesis applications.

## 1. Introduction

In circuit theory, the three fundamental linear passive elements are the resistor, capacitor, and inductor. These components are defined by their constitutive relationships; a resistor governs the voltage–current relationship, an inductor describes the current-flux linkage, and a capacitor establishes the charge–voltage relationship. However, prior to 1971, no element formally linked charge (q) and magnetic flux (φ). This gap was addressed when Leon O. Chua introduced the existence of a fourth fundamental nonlinear element, the memristor, which defines a direct relationship between charge and flux [1,2]. The general features of the memristive system, in which the memristor’s resistance depends on a collection of state variables, were deduced by Chua and Kang [3]. For a nonlinear device to be classified as a memristor, it must satisfy three defining characteristics [4]. First, when driven by a bipolar periodic signal, it must exhibit a pinched hysteresis loop in the voltage–current plane. The second requirement is that as the excitation frequency rises above a critical value, the area of this hysteresis loop must decrease monotonically. Finally, at sufficiently high frequencies, the hysteresis loop must collapse into a single-valued function, indicating frequency-dependent behavior.

Memristive systems are an important development of the original memristor idea, which was first introduced by Chua and later developed into a wider range of memory-based circuit elements. These devices demonstrate distinctive electrical characteristics, including hysteresis curves that pass through the origin while maintaining state-dependent resistance properties without energy storage capabilities. A significant breakthrough occurred in 2008 when HP Laboratories successfully fabricated the first physical realization of a memristor device [5]. Even though many ways to make memristive parts have worked well, making small, reliable solid-state versions is still very challenging and costly. These manufacturing difficulties have currently restricted widespread commercial adoption of physical memristor devices. In response to these practical limitations, researchers have developed various circuit-based approaches to emulate memristive behavior. Recent work has particularly focused on current-mode implementation techniques due to their advantageous operational characteristics [6,7,8,9]. Such work has led to the utilization of multiple active circuit building blocks in memristor emulator designs, including Differential Voltage Current Conveyor Transconductance Amplifier (DVCCTA) [10], Differential Difference Current Conveyor (DDCC) [11], Voltage Differencing Transconductance Amplifier (VDTA) [12], Voltage Differencing Current Conveyor (VDCC) [13], and Second-Generation Current Conveyor (CCII) [9,14,15].

As memristors possess the capability to store both digital and analog data in an easy-to-use and power-efficient manner, they are commonly utilized in digital memory, logic circuits, biological systems, analog processing circuits, and analog in-memory computing [16]. For neural network use, a memristor model made from tantalum oxide that can be adjusted more easily was studied in [17]. In [18], Mladenov presented several applications for memristors, one of which was the hybrid memory method. Furthermore, Refs. [19,20] devote a significant amount of attention to discussing an additional application perspective of memristor emulators. However, most of these circuits exhibit limitations, including high power consumption, restricted frequency response, and constraints in their operational modes (e.g., grounded configurations). The energy usage of active components represents a considerable limitation, particularly in extensive neuromorphic systems where energy efficiency is essential.

Creating easier, more efficient, and faster memristor emulators is still a big research challenge, especially for new uses in neuromorphic computing and fast signal processing. Current investigations focus on innovative circuit architectures to overcome existing performance limitations. Recent studies on passive memristor emulators [21] show promising benefits, such as needing less power and having simpler designs because they use fewer active components. Notable implementations include a floating memristor emulator [22] constructed with just three NMOS transistors, a grounded capacitor, and a current source. While this configuration offers design simplicity, it exhibits constrained behavioral control and a restricted operational frequency ceiling of 13 MHz. Another approach [23] presents a grounded memristor emulator capable of reaching 100 MHz operation frequencies, employing three MOS transistors and a grounded MOS capacitor. However, this design suffers from a critical limitation—the absence of post-fabrication tuning capability, which significantly reduces its versatility across different application scenarios.

This paper presents a novel memristor emulator design based on an operational transconductance amplifier (OTA), specifically tailored for neuromorphic medical applications. The proposed circuit replicates key memristive properties while offering enhanced performance for biomedical implementations. A critical application of this emulator is its integration into a Memristive Integrate-and-Fire (MIF) neuron circuit, which accurately mimics biological neuronal behavior. The MIF circuit generates adaptive spiking signals whose frequency varies with input voltage and pulse width, an essential feature for realistic neural simulation. With operational capabilities spanning nanosecond to microsecond ranges, this design shows significant potential for advanced neurological interfaces, particularly in retinal prosthetics [24]. Retinal implants represent a transformative application of this technology. These devices restore partial vision to patients with degenerative retinal conditions, such as age-related macular degeneration or retinitis pigmentosa, by electrically stimulating surviving retinal neurons. The compatibility of our memristor emulator with these biomedical systems is noteworthy.

The remainder of this paper is structured as follows. Section 2 details the proposed emulator’s architecture, comprising a CMOS inverter and OTA, along with its mathematical modeling. Section 3 analyzes simulation results of the OTA-based core circuit. Section 4 demonstrates its implementation in a medical use case, emphasizing neuromorphic functionality. Section 5 concludes with key findings and future directions.

## 2. Design of the Emulator

### 2.1. Characteristics

Memristors exhibit two fundamental control mechanisms based on their operational characteristics. These devices can be governed either by electrical charge or magnetic flux, leading to distinct behavioral classifications. In charge-controlled memristors, the magnetic flux (φ) depends solely on the accumulated charge (q), expressed mathematically as φ(t) = f(q(t)). Conversely, flux-controlled memristors demonstrate the inverse relationship, where the charge becomes a function of the applied flux: q(t) = f(φ(t)) [25]. Research indicates that optimal memristor operation depends on these control paradigms [26]. For charge-controlled devices, the voltage–current relationship follows specific characteristic equations (Equation (1)), while flux-controlled variants obey different governing principles (Equation (2)). This fundamental distinction determines how memristors respond to electrical stimuli and store state information, which is crucial for their application in memory devices and neuromorphic systems.

*v* = *M*(*q*) × *i* (1)

*i* = *w*(*φ*) × *v* (2)

Here, *i* is the memristor current, *v* is the voltage, *q* is the charge, and *φ* is the flux. *M*(*q*) is the memristance and w(*φ*) is the memconductance and are defined as Equation (3).

(3) $$M\left(q\right)=\frac{1}{w(\varphi )}$$

### 2.2. Circuit Description and Mathematical Modeling

**Figure 1:**
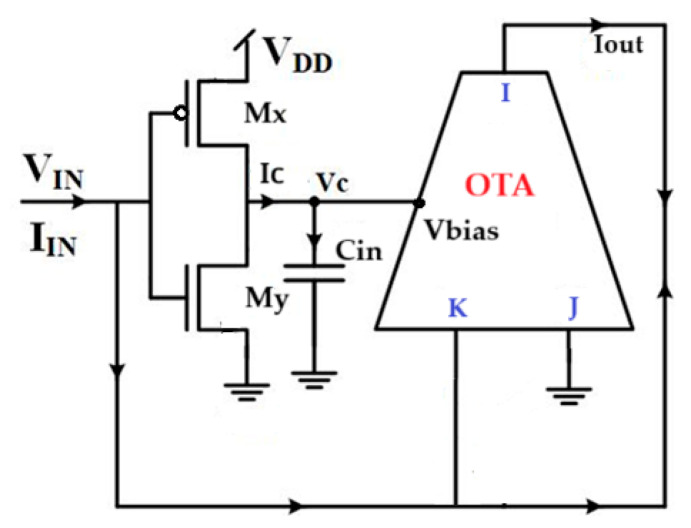
Proposed emulator topology.

In this work, the proposed memristor emulator uses an operational transconductance amplifier (OTA) as its core current-mode component. As shown in Figure 1, the OTA integrates with a CMOS inverter and memory-state capacitor to emulate a complete memristive system, replicating physical memristor characteristics with enhanced tunability and integration potential.

The OTA serves as the circuit’s core element, functioning as a voltage-controlled current source. Its operation centers on the transconductance parameter ($g_{m}$), which directly converts the differential input voltages ($V_{J}$ and $V_{K}$) into a proportional output current ($I_{out}$). This critical voltage-to-current conversion, mathematically defined by Equation (4), enables the emulator to faithfully reproduce memristive behavior. The g_m_ value not only determines the conversion efficiency but also influences the dynamic response of the entire memristor emulation system.

(4) $$I_{out}=gm\;(V_{J}-V_{K})$$

With

(5) $$gm=\frac{K^{\prime }}{\sqrt{2}}(V_{bias}-2V_{th})$$

In which $K^{\prime }$ is a parameter of the MOS device and $V_{th}$ is the threshold voltage. From Vbias, the OTA obtains information on external tunability.

In this architecture, input ports I and K of the OTA are connected to the CMOS inverter’s input terminal, while input port J is grounded. This configuration ensures the memristor emulator operates in a grounded topology. Furthermore, only one power source was needed to achieve the suggested memristor model compared to many other models as presented in [27]. However, we may achieve both incremental and decremental memductance by merely switching the connections of the input terminals using a switch, which is a noteworthy characteristic of this model in grounded arrangement.

The memristor emulator’s incremental operation reveals an important voltage distribution pattern. The input voltage ($V_{in}$) appears simultaneously at three critical nodes: the gate terminals of both $M_{x}$ and $M_{y}$ transistors and the K terminal of the OTA. This shared voltage configuration creates a synchronized control mechanism for the entire circuit. The transconductor stage, implemented through the $M_{x}-M_{x}$ transistor pair, generates an output current that follows the relationship defined in Equation (6). This current represents the fundamental conversion of the input voltage into the circuit’s memristive response. The precise mathematical formulation in Equation (6) captures the essential nonlinearity that enables the emulator to reproduce authentic memristor behavior.

(6) $$I_{c}={gm}_{xy}V_{IN}(t)$$

${gm}_{xy}$ denotes the transconductance gain of the transconductor stage.

A voltage $V_{C}$, equal to the bias voltage $V_{bias}$, is developed across the capacitor $C_{in}$ by the drain current flowing via transistors $M_{x}$ and $M_{x}$. Because of the high gate input impedance of $V_{bias}$ terminal, there is no current flowing to it.

The current enters the capacitor (*C*) and generates a potential across it, represented by Equation (7):

(7) $$V_{c}=V_{bias}=\;\frac{{\;gm}_{xy\;}\varphi_{IN}(t)}{c_{in}}$$

The OTA generates its output current through a straightforward yet crucial operation: the product of its transconductance gain (*g*_m_) and the voltage at its K terminal. This output current exhibits an important characteristic; it maintains equal magnitude but opposite polarity to the input current ($I_{IN}$). This complementary relationship can be expressed as

(8) $$I_{out}=-gmV_{IN}=-I_{IN}$$

The proposed memristor emulator’s memductance (*W*) is mathematically defined by Equation (9), which captures its essential behavior. This key parameter exhibits continuous tunability, with its value directly determined by the voltage across capacitor $C_{in}$. As the capacitor voltage varies, the memductance transitions smoothly through multiple adjustable states, effectively creating a spectrum of programmable resistance levels. This continuum of operational states enables precise control over the emulator’s resistive characteristics, making it particularly suitable for applications requiring analog memory or neuromorphic computation.

(9) $$w\left(\varphi \right)=\frac{I_{IN}}{V_{IN}}=-K^{\prime }\left(2V_{th}\right)+\frac{{\;K\prime gm}_{xy\;}\varphi_{IN}(t)}{\sqrt{2}C_{in}}$$

Additionally, the derived memductance expression follows standard flux-controlled memristor characteristics, as shown in Equation (9). This equation contains both time-invariant and time-variant components, revealing how memductance depends on charge carrier mobility in the channel. Notably, this mobility parameter exhibits temperature dependence, similarly to the well-documented behavior of HP’s TiO_2_ memristor [5].

When voltage is applied to the circuit, the capacitor begins accumulating charge through a process governed by two key factors: the circuit’s inherent resistance and the magnitude of the applied voltage. These parameters determine the charging rate, with higher voltages and lower resistances enabling faster charge accumulation. The voltage across the capacitor develops gradually as the circuit integrates the flowing current over time. This charging mechanism serves a dual purpose in memristor emulation. By dynamically adjusting the effective resistance in response to the charging state, the circuit successfully reproduces the memristor’s characteristic resistance fluctuations that depend on voltage history. The continuous current integration through the capacitor naturally mimics the memristor’s ability to “remember” its charge history, a fundamental property that distinguishes memristors from conventional circuit elements. The charge storage in the capacitor produces a direct observable effect; it generates the distinctive pinched hysteresis loop that serves as the fingerprint of memristive behavior. This looping characteristic emerges precisely because the circuit’s resistance state depends on the accumulated charge history, creating the memory effect that is central to memristor operation.

### 2.3. OTA Design

**Figure 2:**
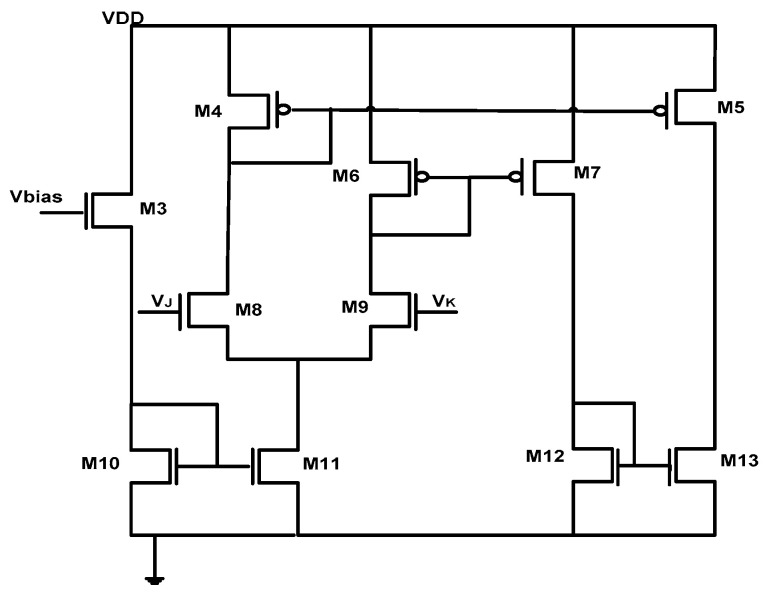
OTA transistor level circuit.

Figure 2 illustrates the internal structure of the OTA used in our design. The circuit implementation utilizes 11 transistors, all carefully biased in their saturation regions to ensure optimal performance. This configuration enables precise voltage-to-current conversion through the amplifier’s transconductance property. The OTA’s circuit functionality involves converting the differential input voltage ($V_{J}-V_{K}$) into a proportional $I_{out}$. This conversion is governed by the gm, which serves as the critical proportionality factor between input voltage and output current. The $V_{Bias}$ provides control over the gm value, allowing for adjustable amplifier sensitivity to meet different application requirements.

**Figure 3:**
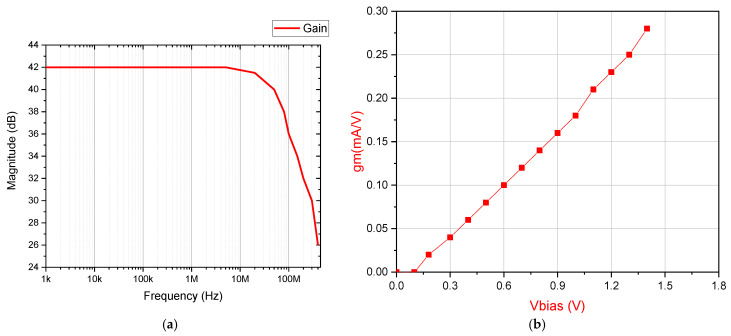
(**a**) OTA frequency response. (**b**) OTA transconductance versus Vbias simulation result.

The proposed circuit was evaluated through detailed simulations conducted in a standard analog design environment. All tests were performed under nominal conditions using a 1.2 V power supply, representing typical operating parameters for modern integrated circuits. The transconductance amplifier’s frequency response characteristics are presented in Figure 3a, demonstrating stable operation across the relevant frequency spectrum. Figure 3b illustrates the linear relationship between $V_{Bias}$ and gm, with $V_{Bias}$ varied from 0 to 1.8 V. This proportional dependence, which aligns perfectly with Equation (5), provides precise control over the amplifier’s conversion characteristics.

The simulation results demonstrate that the proposed design achieves robust performance across several critical parameters. A phase margin of 64° ensures stability under varying load conditions, while a fast 2.5 V/μs slew rate enables quick signal transitions. The amplifier’s 22.9 MHz unity-gain bandwidth makes it well-suited for medium-frequency applications, and its high 89.5 dB common-mode rejection ratio effectively suppresses noise interference. These characteristics collectively make the design highly suitable for memristor emulation in neuromorphic computing, where precise analog behavior and reliability are essential. Furthermore, the circuit’s linear tunability and strong electrical performance position it as a versatile solution for adaptive and reconfigurable systems.

## 3. Results and Discussion

### 3.1. Simulation Results

**Figure 4:**
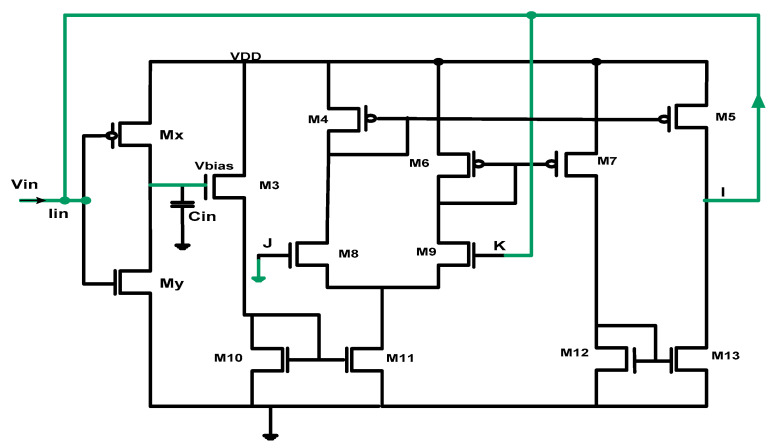
Memristor emulator architecture.

Figure 4 presents the complete schematic of the proposed memristor emulator, implemented in 180 nm TSMC technology. Operating at 1.2 V supply voltage, the compact design incorporates just 13 transistors while maintaining the remarkably low power consumption of only 0.1 mW. The transistor sizing parameters, critical for achieving optimal performance, are systematically detailed in Table 1, which specifies the aspect ratios (W/L) for all devices in the design. This careful transistor-level optimization enables the circuit to accurately emulate memristive behavior while meeting power efficiency requirements for practical applications.

**Figure 5:**
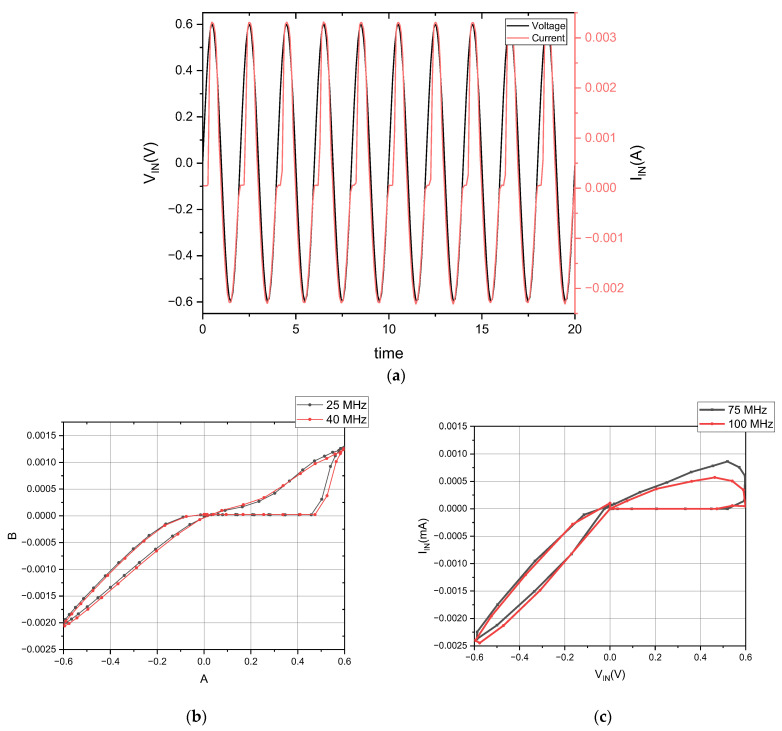
(**a**) Transient curve simulation. (**b**) PHL at 25 MHz and 40 MHz. (**c**) PHL at 75 MHz and 100 MHz.

The fundamental behavior of memristors manifests through a characteristic pinched hysteresis loop (PHL) when driven by sinusoidal signals. To thoroughly examine this frequency-dependent phenomenon, we applied a 600 mV sinusoidal input while sweeping across multiple frequency points. The emulator’s circuit includes a 3 pF capacitor that enables proper memristive operation. The simulation results clearly demonstrate the emulator’s dynamic behavior under varying operating conditions. As shown in Figure 5a, the circuit maintains excellent stability up to 40 MHz, exhibiting consistent transient responses across multiple cycles. The frequency-dependent characteristics are further illustrated in Figure 5b,c, which reveal how the PHL shape systematically varies with changing input frequency. These results validate the circuit’s ability to reproduce essential memristor characteristics while maintaining consistent operation across different frequencies.

**Figure 6:**
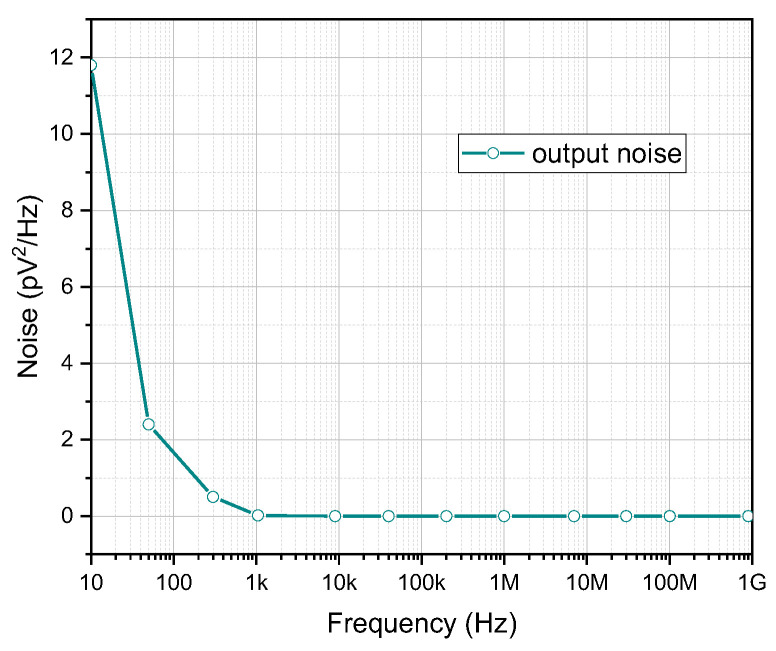
Output noise characteristic of the memristor emulator circuit.

To understand the main noise sources in the CMOS-based memristor emulator design, which mainly come from flicker and channel thermal noises, the total output noise has been calculated over a large frequency band as shown in Figure 6. Hence, for frequencies between 10 and 100 Hz, the noise level (highest average noise power), is expected to be between 11.8 pV^2^/Hz and 1.02 pV^2^/Hz. However, the noise is very low for frequencies from 1 kHz to higher MHz. Nonetheless, the noise is minimal for the frequency range of 1 kHz to 1 GHz. The effect of noise is negligible throughout the memristor’s operating range, which is centered around 20 MHz to 100 MHz per unit of bandwidth, as shown in Figure 6. One obvious benefit of the design is that the circuit has an excellent signal-to-noise ratio (SNR).

**Figure 7:**
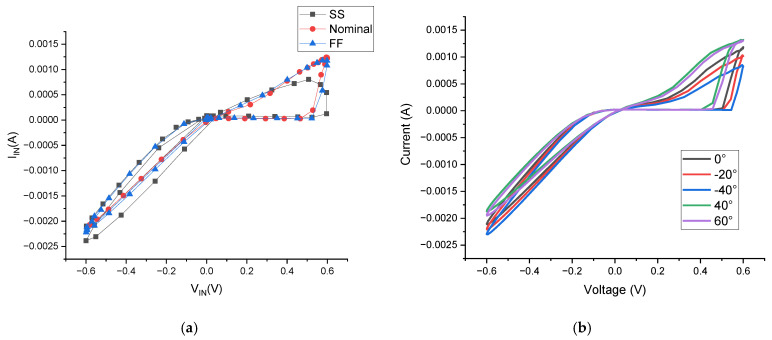
(**a**) Corner analysis. (**b**) HPL at different temperatures.

Since the size of MOSFETs and other components used in circuit simulation can differ from their actual sizes due to manufacturing process variations, it is essential to perform process corner analysis. This analysis guarantees the resilience of the MOSFETs in the design against process variation and to ensure that performance remains within the slow and fast corners. Figure 7a demonstrates the circuit’s performance across different process corners, evaluating worst-case scenarios that include slow (SS) and fast (FF) transistor characteristics alongside nominal conditions. The FF corner produces peak current values while the SS corner yields minimum currents, with both extremes exhibiting only minor deviations from nominal operation, confirming the design’s robustness against fabrication variations.

Furthermore, the circuit’s thermal stability was verified through extensive temperature testing from −40 °C to +60 °C, as shown in Figure 7b. Operating at 40 MHz, the memristor emulator exhibits predictable thermal behavior; current levels rise proportionally with temperature while maintaining the essential pinched hysteresis characteristics. Importantly, the fundamental memristive properties remain stable across the entire temperature range, demonstrating reliable operation in diverse environmental conditions. These results validate the design’s suitability for practical implementations where process and temperature variations are significant concerns.

**Figure 8:**
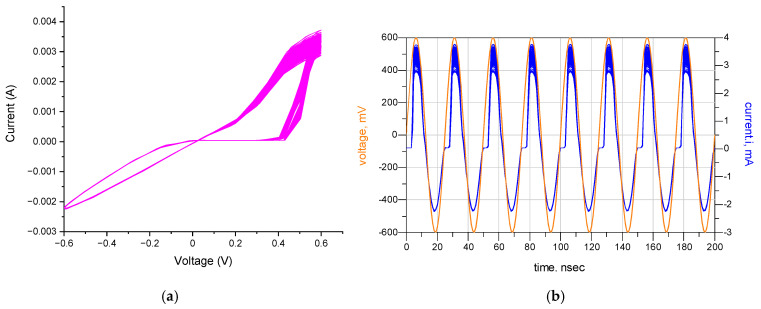
Monte Carlo simulation: (**a**) transient curve and (**b**) V–I characteristic.

The current flowing through the designed memristor may deviate from the desired level as a result of variations in transistor parameters. Monte Carlo analysis serves as an essential method for assessing the influence on memristor current resulting from the variation in transistor parameters. Consequently, a Monte Carlo analysis was performed, considering variations in process and mismatch parameters, to illustrate the potential fabrication deviation of the proposed emulator circuit. The responses were derived from conducting Monte Carlo simulations 200 times. The analysis of the memristor circuit in Figure 8 indicates that the fluctuation in memristor current is confined to 0.7 mA, which corresponds to merely a 17% deviation from the nominal value of 4 mA. This illustrates the robustness of the circuit design, even when components show minor deviations within acceptable tolerance levels. It is important to highlight that, despite these variations, the memristor functions within acceptable parameters, maintaining the overall stability and reliability of the circuit under different process conditions. This underscores the strength of the design and its capacity to uphold reliable performance even in the face of slight variations in components.

**Figure 9:**
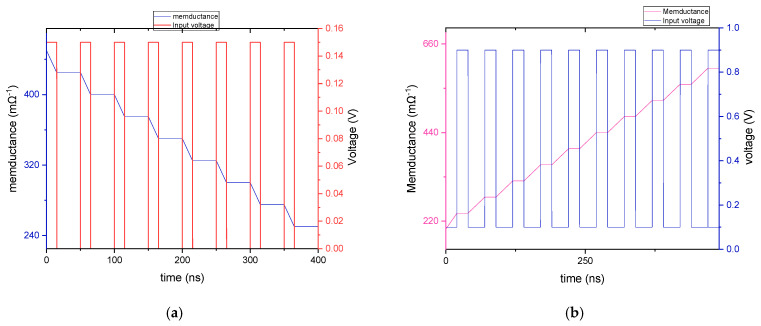
(**a**) Non-volatility (decremental). (**b**) Non-volatility (incremental).

The proposed memristor emulator demonstrates reliable non-volatile characteristics, maintaining its resistance state without external power. When stimulated by input signals, the circuit smoothly transitions between different resistance states while preserving each state until the next activation. Our simulations used 150 mV input pulses with a pulse width of 15 ns and a period of 50 ns to evaluate this behavior. Figure 9 demonstrates several critical aspects of the memristor emulator’s behavior. The memductance transitions occur in distinct, quantized steps, producing a characteristic staircase pattern in the waveform. Between input pulses, the memductance value remains perfectly stable, clearly exhibiting the non-volatile nature of the device. Most significantly, the circuit retains its state indefinitely even in the absence of any external input signals, confirming its ability to function as a reliable memory element. Figure 9a,b further demonstrate the emulator’s capability to achieve both incremental and decremental memductance states over time. This multi-state operation, combined with non-volatile storage, makes the design particularly suitable for neuromorphic applications. Specifically, it can effectively store synaptic weights in neural network implementations, where precise analog memory and gradual state transitions are essential for learning algorithms and adaptive processing.

### 3.2. Comparison

This work introduces a novel memristor emulator design operating across a 20 MHz to 10 0 MHz frequency range, implemented in 180 nm CMOS technology. The circuit employs an efficient grounded configuration requiring only thirteen transistors, making it particularly compact compared to existing solutions. The proposed design demonstrates several competitive advantages, as detailed in Table 2′s comprehensive comparison with state-of-the-art emulators. Key comparison metrics include maximum operating frequency, circuit topology, fabrication technology, and power efficiency.

The comparative analysis in Table 2 demonstrates significant improvements offered by our design over existing memristor emulators. Key advantages include the following:

- Low Power Consumption: The proposed emulator consumes only 0.1 mW, outperforming all listed works such as 8.74 mW in [28], 1.34 mW in [29], 9.567 mW in [15], and 3.87 mW in [33].
- High-Frequency Performance: The proposed emulator achieves a high operating frequency of 100 MHz, matching the highest performance reported in the literature [32], while offering significant architectural improvements. Unlike the reference design requiring 15 MOS transistors and a differential voltage current conveyor (DVCC) with three additional MOS devices, our implementation attains this high-frequency operation with greater efficiency, utilizing only 13 transistors in total and a simpler configuration based on a single OTA with just two MOS components.
- Compact and Efficient Design: The proposed design uses only 13 transistors, fewer than most reported works: 27 in [28], 24 in [15], 32 in [30], 34 in [33], and 22 in [34]. Additionally, it requires just one active block (OTA) and one capacitor, reducing passive component count compared to designs using resistors like [28,30,33].
- Power Efficiency: The design achieves enhanced power efficiency through three key features: a simplified circuit implementation that minimizes both static and dynamic power consumption; avoidance of DC offset circuitry, as transistor polarization is maintained in the required operating region through careful biasing; and adoption of an asymmetric power supply scheme (0 V to 1.2 V) to further optimize energy efficiency. These architectural choices collectively enable significant power savings while preserving the circuit’s memristive functionality.

In summary, the proposed emulator has been developed with explicit attention to scalability requirements. The current implementation uses low transistors numbers (13 transistors) per memristor emulator in 0.18 μm CMOS technology, achieved through careful transistor-level optimization and minimal component count compared to similar published works. Power management remains a critical consideration for large-scale implementations, where each emulator unit is measured to consume only 100 μW during active operation. However, as array dimensions expand, the cumulative power demand must be carefully addressed. Power-gating techniques are being considered for future implementations to effectively manage system-wide energy consumption. The interconnect challenge emerges as the most significant scalability limitation, where routing complexity grows substantially with array size. Architectural solutions employing hierarchical structures are anticipated to be implemented to maintain system manageability. While full-scale integration studies have not yet been conducted, these operational trade-offs are recognized as essential design factors. Future investigations will be directed toward comprehensive layout analysis and system-level power simulations to quantitatively evaluate these parameters.

Additionally, our CMOS-based memristor emulator offers distinct advantages in modeling accuracy and controllability compared to physical memristors, providing deterministic and repeatable memristive behavior through well-defined transistor parameters while avoiding the stochastic switching and process variations characteristic of nanoscale devices. The design achieves continuous analog memory capability through precise capacitor-based state control, supporting tunable frequency states (20–100 MHz range) that closely approximate real memristor dynamics while offering superior reproducibility compared to digital emulator alternatives. The dynamic response is configurable via capacitor tuning, enabling the flexible emulation of diverse neuromorphic behaviors, from short-term plasticity to long-term potentiation. However, these benefits come with inherent CMOS trade-offs, including greater area/power overhead than physical memristors and volatile state retention. To address device variability, we employ differential topologies to minimize CMOS mismatch effects, while our shared biasing scheme enhances scalability for medium-sized arrays. The design intentionally incorporates nonlinear dopant drift and asymmetric switching characteristics through controlled state transitions, providing more realistic emulation than simplified linear models. These features position our emulator as particularly suitable for prototyping noise-tolerant neuromorphic systems where predictability and tunability outweigh the density advantages of physical memristors.

Although traditional memory technologies like SRAM, DRAM, and Flash exhibit high speed and mature fabrication processes, they lack the non-volatility and analog tunability essential for in-memory computing tasks such as neuromorphic processing. Moreover, as highlighted in [35,36], physical memristors offer high density and non-volatility but suffer from variability, limited endurance, and fabrication challenges, particularly in analog computing applications. While not non-volatile in the strict sense, our emulator provides an analog memory-like behavior with fully controllable and repeatable characteristics. It allows prototype in-memory computing architectures while avoiding the fabrication uncertainties associated with emerging memory technologies.

## 4. Application: MIF Neuron Circuit for Retinal Protheses

### 4.1. Memristive Integrate and Fire (MIF) Neuron Circuit

**Figure 10:**
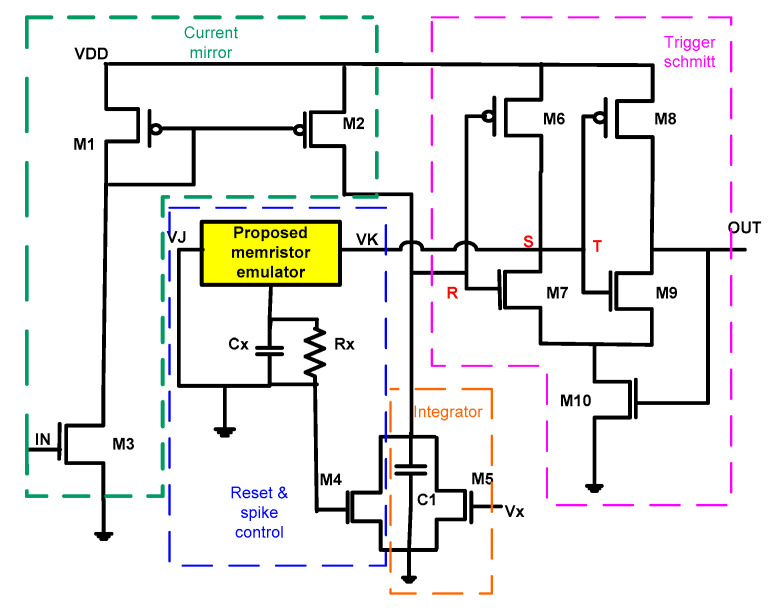
MIF neuron circuit architecture.

The proposed MIF neuron design innovatively enhances the conventional LIF (Leaky Integrate-and-Fire) neuron model [37] by incorporating memristive technology. While the traditional LIF circuit effectively mimics biological neuron behavior through its four core components—spike control unit, integration stage, current mirror, and Schmitt trigger—our modified architecture introduces a crucial advancement by replacing the standard reset mechanism with a memristor emulator. This strategic modification enables the MIF neuron to achieve more biologically realistic spiking patterns while supporting continuous resistance state transitions, as illustrated in Figure 10. The integration of memristive properties provides the circuit with dynamic memory capabilities, allowing it to better replicate the adaptive nature of biological neurons. This enhanced functionality makes the MIF neuron particularly valuable for neuromorphic computing applications that require both precise temporal signal processing and analog memory retention. The design maintains the structural simplicity of the original LIF model while adding the critical dimension of memristive state modulation, creating a more versatile and functionally rich neuron implementation.

The MIF neuron’s spiking behavior initiates when an input pulse activates the gate of the transistor M3, triggering the current mirror to inject charge into the integration stage. As capacitor C_1_ accumulates this current, it generates a rising membrane potential voltage that mimics biological neuronal depolarization. This voltage continues building until reaching the Schmitt trigger’s threshold voltage V_x_*,* set by the transistot M5, causing node R to switch high and generate an output spike at the OUT terminal. The spike generation simultaneously drives nodes S and T low, creating a feedback loop that (1) lowers the memristor emulator’s input V_k_, (2) charges reset capacitor C_x_ to VDD, and (3) activates transistor M4 to rapidly discharge the membrane potential. This complete cycle—integration, threshold detection, spike generation, and reset—closely emulates the fundamental process of biological neuronal firing while incorporating the memristor’s state-dependent properties for enhanced functionality.

The MIF neuron circuit incorporates a biologically inspired refractory period mechanism through the RC network formed by resistor R_x_ and capacitor C_x_. During this recovery phase, C_x_ discharges through R_x_ with a time constant τ = R_x_C_x_ that governs three key aspects of neuronal behavior: the interval between consecutive spikes, called refractory period, the maximum firing frequency, and the circuit’s recovery time. This refractory period mimics the absolute refractory phase in biological neurons, during which the circuit remains unresponsive to subsequent input pulses. The spike frequency can be precisely modulated by adjusting either the integration capacitor C_1_, which controls the charging rate of the membrane potential, or the reset capacitor C_x_, which determines the discharge rate. This tunability allows the circuit to emulate various neuronal firing patterns while maintaining autonomous operation without requiring external reset signals. The complete biological analogy is achieved through these carefully designed dynamics, with all critical implementation parameters detailed in Table 3.

### 4.2. Adaptive MIF Neuron for Artificial Retina Design

A retinal prosthesis is a biomedical device designed to restore partial vision to people who have lost their sight due to degenerative retinal diseases like retinitis pigmentosa or age-related macular degeneration. These conditions typically damage the photoreceptors (rods and cones) but spare the inner retinal neurons (like ganglion cells), which can be electrically stimulated. Retinal implants bypass the damaged photoreceptors by directly stimulating the remaining functional cells in the retina or the optic pathway. Such device can either be used as a medical application or for biomimetic machine vision devices which require features such as an artificial retina for picture collection and processing, as well as an artificial network that mimics the cortex for visual inspection and identification tasks [38].

**Figure 11:**
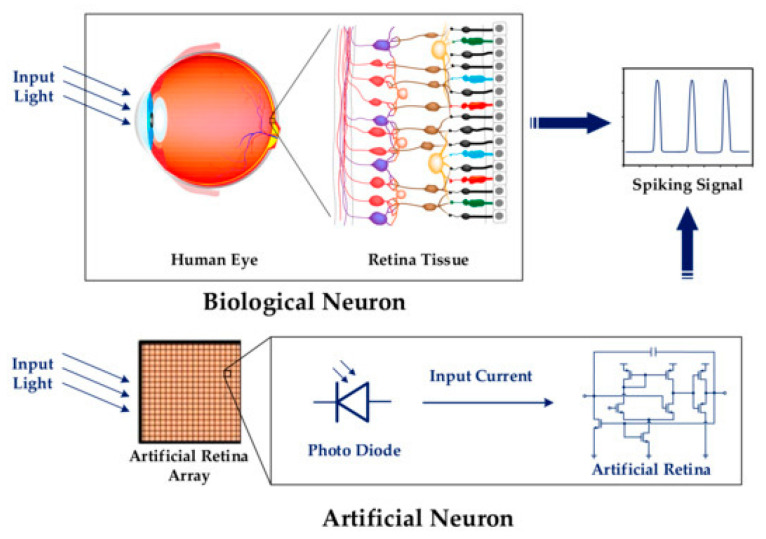
Schematic representation of the operational mechanism of artificial neurons that emulate the biological retina.

The retina contains many photosensitive neurons that perceive light signals and produce distinct spike patterns in response to diverse conditions, such as bright or dim environments or transitions from light to darkness [39,40]. Figure 11 presents the biological vision system. Different types of light can affect the retina in different ways thanks to horizontal cells and photoreceptor cells (cones and rods). Visual adaptation makes it possible to see things clearly in a range of lighting conditions, from bright to dim. After the retina’s adaptive process, an overexposed picture can be turned into a normal picture with no big changes in light for the brain. The horizontal cells tuning rods and cones make the retina adaptable. If you change the threshold of the retina, the frequency of biological spikes to the brain would also change.

Prior studies have shown that the Hodgkin–Huxley neuron circuit is capable of producing up to 23 distinct biological neuron-spiking behaviors, indicating its significant biomimetic properties [41]. However, the absence of adaptive properties in the memristor device results in numerous independent and unconvertible spiking behaviors, thereby constraining the implementation of Hodgkin–Huxley circuits. The proposed circuit has the potential to render spiking models convertible and interdependent, allowing for transitions from tonic spiking or bursting to quiescent states or oscillations through the utilization of adaptive MIF circuits.

In the design, the memristor emulator is integrated within the adaptive feedback loop of the MIF neuron circuit, where it modulates the feedback resistance dynamically based on the membrane potential. This modulation directly influences the inter-spike interval, allowing the neuron’s firing frequency to adapt to varying input currents that emulate changing light conditions in retinal prostheses. The emulator’s resistance state adjusts in real time, effectively implementing a form of spike-frequency adaptation similar to biological neurons. While the present work emphasizes transistor-level simulations, we acknowledge that full system-level validation under variable luminance patterns, including photodiode interfacing and noise resilience, will be addressed in future experimental prototypes.

### 4.3. Results and Discussion

**Figure 12:**
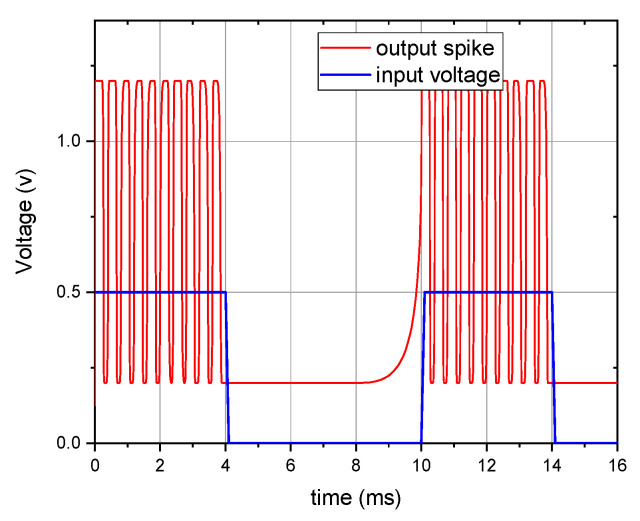
Spiking signal output of the MIF circuit.

The circuit’s operation was validated through comprehensive simulations of both the leaky integration behavior and refractory period dynamics. Using a 500 mV input pulse with 10 μs period and 40% duty cycle, we observed the complete spiking cycle as depicted in Figure 12. The system demonstrates three distinct operational phases: (1) leakage phase—in the absence of input pulses, the output node shows characteristic leakage current, mimicking biological neuron membrane properties; (2) integration phase—when stimulated, the circuit integrates the input signal through capacitor C_1_ (30 pF), gradually building membrane potential; and (3) firing phase—upon reaching the precisely set 0.45 V threshold (V_x_), the neuron generates an output spike and initiates the refractory period through capacitor C_x_ (50 nF). These simulations confirm the circuit’s ability to faithfully reproduce key neuronal characteristics, including threshold-controlled spike generation, leaky integration behavior, refractory period dynamics, and precise temporal response to input stimuli. The results demonstrate successful emulation of biological neuron functionality while maintaining consistent operation across multiple spiking cycles.

**Figure 13:**
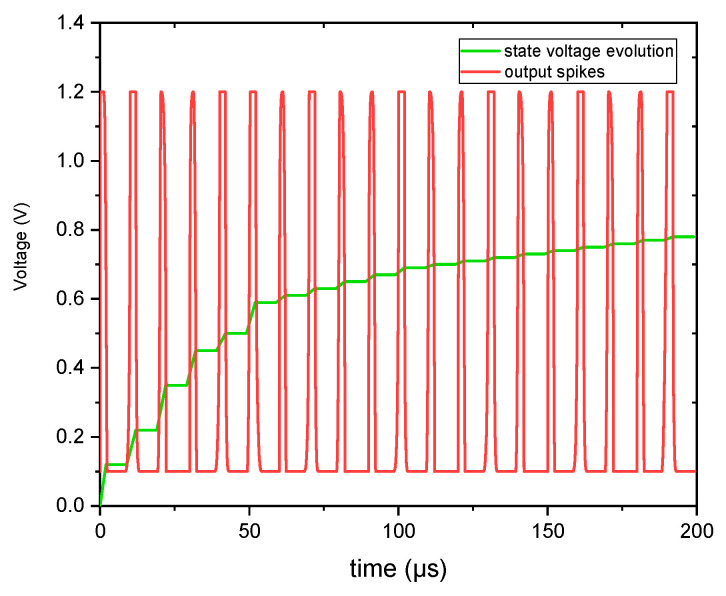
MIF neuron exhibiting a spiking response alongside the corresponding state voltage indicating varying state voltages for each output spike.

The MIF neuron’s design demonstrates a unique self-regulating mechanism where capacitor C_x_ autonomously controls both spike termination and refractory period initiation through its charge–discharge cycles. These electrical transitions directly influence the memristor’s memductance, creating a dynamic memory effect; charging establishes new conductance states while discharging preserves previous ones, as clearly shown in Figure 13. This intrinsic behavior mimics the human brain’s efficient architecture where computation and memory coexist, enabling the neuron to perform in-memory processing without external control. The circuit’s ability to organically integrate signal processing with state retention through these coupled capacitor–memristor interactions represents a significant advancement in neuromorphic engineering, closely replicating biological neurons’ functional principles while maintaining compact, energy-efficient operation.

Figure 13 illustrates that each spike corresponds to a specific Cx voltage or state voltage at precise moments. These various state voltages indicate different memductance states. Consequently, the MIF neuron circuit-based emulator can showcase a range of tunable memductance states and produce spiking output.

## 5. Conclusions

In this paper, a resistorless, tunable grounded flux-controlled memristor emulator was designed. An operational transconductance amplifier (OTA) is utilized, functioning as the active component alongside two MOS transistors. The emulator paradigm is clear and appealing. The OTA input port, when reversed, functions effectively in both incremental and decremental configurations. Simulator testing effectively supported theoretical investigation. Multiple simulations and analyses were performed to assess the resilience of the proposed circuit. The robustness of both the corner and Monte Carlo simulations has been assessed and confirmed satisfactorily. The memristor emulator is designed to function at frequencies up to 100 MHz while consuming 0.1 mW of power, utilizing 180 nm CMOS technology. The simulation results indicate that the design exhibits favorable noise performance. The MIF neuron circuit has been designed to show the potential applications of the proposed emulator circuit, especially in retinal protheses architecture.

Future work will include fabrication and experimental characterization of the memristor emulator circuit to validate its performance under real-world operating conditions. Key aspects to be addressed include process variations, power consumption, and area efficiency.

## Figures and Tables

**Table 1:** Aspect ratios of the OTA MOSFETs.

MOSFET	W/L (µm)
Mx	3/0.18
My	0.5/0.18
M3	30/0.18
M4	36/0.18
M5	150/0.18
M6	3.6/0.18
M7	100/0.18
M8	160/0.18
M9	90/0.18
M10–M11	5/0.18
M12–M13	7/0.18

**Table 2:** Comparison of the proposed memristor emulator circuit with existing design.

Ref	No. of Active Components	No. of Passive Components	Power Supply	Technology Used	No. of MOS	Power Consumption	Max. Operating Frequency
[28]	1-DVCCTA	R-1 C-2	±1 V	180 nm	27	8.74 mW	12.8 MHz
[29]	2-VDIBA	C-1	±1 V	180 nm	18	1.34 mW	12.7 MHz
[15]	1-CCII, 1-OTA	R-1 C-1	±1.2 V	180 nm	24	9.567 mW	26.3 MHz
[30]	1 VDTA, 1 MULT	R-2 C-1	±0.9 V	180 nm	32	N/A	2 MHz
[31]	1 VDTA	1 R 1 C	±0.9 V	180 nm	16	N/A	50 MHz
[32]	1 DVCC, 3-MOS	C-1	±1.25 V	180 nm	15	N/A	100 MHz
[33]	1-OTA, 1-MO OTA, 1-Analog amplifier	R-1 C-2	±1.25 V	180 nm	34	3.87 mW	1 MHz
[34]	1 VDCC	M_R_-1 C-1	±0.9 V	180 nm	22	N/A	700 KHz
This work	1-OTA, 2-MOS	C-1	1.2 V	180 nm	13	0.1 mW	100 MHz

**Table 3:** MIF circuit parameters.

Parameter	W/L (µm)
M1-M2	0.4/0.18
M3-M7	0.28/0.18
M4-M6-M8-M9	0.25/0.18
M10	0.22/0.18
Rx	10 kΩ

## Data Availability

Data are contained within the article.
